# Retraction: Bacteria-Induced Dscam Isoforms of the Crustacean, *Pacifastacus leniusculus*

**DOI:** 10.1371/journal.ppat.1005630

**Published:** 2016-05-04

**Authors:** Apiruck Watthanasurorot, Pikul Jiravanichpaisal, Haipeng Liu, Irene Söderhäll, Kenneth Söderhäll

After publication of the article, a reader raised concerns about two figures:

- In Figure 5A, lane 1 looks very similar to the top band in lane 7 and lane 2 looks very similar to the top band in lane 5.
- In Figure 7B, in the P/Dscam panel, lanes 1 through 6 all look very similar to each other; in the WWSV VP28 panel, lanes 2 and 3 look very similar to each other; and in the WSSV VP28 panel, lanes 5 through 9 all look very similar to each other.

These concerns were brought to the attention of the Corresponding Author, Kenneth Söderhäll, along with a request to provide the raw blots for each of the two figures to allow for full examination. In response, Dr. Söderhäll informed the journal that the authors were unable to provide the raw blots, stating that the first author, Apiruck Watthanasurorot, who conducted the experiments and who handled and assembled these figures, has been out of contact and he has the original files. A researcher in Dr. Söderhäll’s group, who was not involved in the original study, has repeated the experiments and confirmed the results in Figures 5A and 7B. In addition, other published findings support the conclusions of this paper. However, the authors are retracting this publication due to the possibility of misrepresentation of the data. An investigation of Figure 5A and Figure 7B in this article by Uppsala University, conducted by an Expert Group for Scientific Misconduct at the Central Ethical Review Board, led to the recommendation to retract the article on the grounds of image manipulation by Apiruck Watthansurorot, and the unavailability of the original images.

All of the authors, except Apiruck Watthansurorot who could not be contacted, have agreed to this retraction. The authors apologize to the readers and editors of *PLOS Pathogens* for these errors.

## References

[1] Watthanasurorot A, Jiravanichpaisal P, Liu H, Söderhäll I, Söderhäll K (2011) Bacteria-Induced Dscam Isoforms of the Crustacean, *Pacifastacus leniusculus* . PLoS Pathog 7(6): e1002062 doi: 10.1371/journal.ppat.1002062 21695245 10.1371/journal.ppat.1002062 PMC3111544

